# Supplementary material for: The Evolution and Characterization of the RNA Interference Pathways in Lophotrochozoa
Source: Genome Biol Evol. 2024 May 7;16(5):evae098. doi: 10.1093/gbe/evae098 (PMC11114477; doi:10.1093/gbe/evae098)
Supplement: evae098_Supplementary_Data [file evae098_supplementary_data.zip › Supplementary_materials.zip!/selected_miRNAs/NoGe_selected_miRNAs/mirdeep_pdf_files/NMRB01000064.1_Notospermus_geniculatus_strain_Ushimado_scaffold64,_whole_genome_shotgun_sequence_2363.pdf]

Provisional ID	:	NMRB01000064.1_Notospermus_geniculatus_strain_Ushimado_scaffold64,_whole_genome_shotgun_sequence_2363
Score total	:	48686.2
Score for star read(s)	:	3.9
Score for read counts	:	48681.3
Score for mfe	:	1.6
Score for randfold	:	0
Score for cons. seed	:	-0.6
Total read count	:	95498
Mature read count	:	75055
Loop read count	:	0
Star read count	:	20443

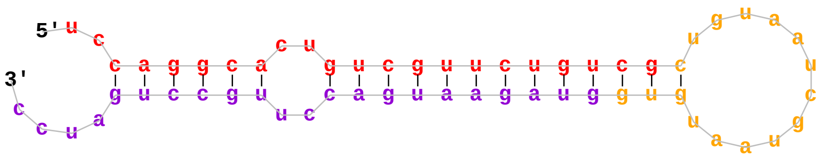

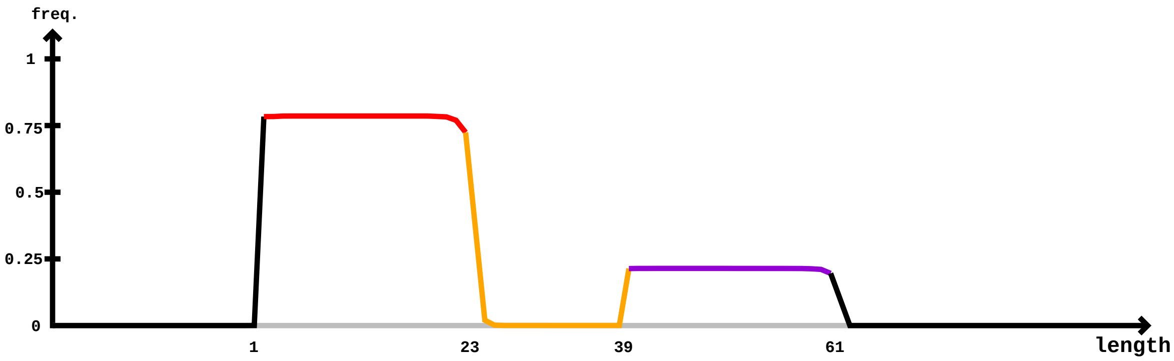


```
                    Mature                              Star
5'- ucacugacggacucguggguuccaggcacugucguucugucgcuguaaucguaaugugguagaaugaccuugccugauccccaagggaacaucaacaucuggacacuugaac   -3'    obs
    ucacugacggacucguggguuccaggcacugucguucugucgcuguaaucguaaugugguagaaugaccuugccugauccccaagggaacaucaacaucuggacacuugaac          exp
    ....(((.(..(((.((((...((((((..((((((((((((...........))))))))))))..))))))...)))).)))..).))).....................   reads    mm    sample
    ....................uccaggcacugucguucu..........................................................................   12       0     Fm1
    ....................uccaggcacugucguucug.........................................................................   10       0     Fm1
    ....................uccaggcacugucguucugA........................................................................   2        1     Fm1
    ....................uccaggcacugucguucugu........................................................................   23       0     Fm1
    ....................uccaggcacugucguucuguc.......................................................................   127      0     Fm1
    ....................uccaggcacugucguucGguc.......................................................................   1        1     Fm1
    ....................uccGggcacugucguucuguc.......................................................................   1        1     Fm1
    ....................uccaggcacuCucguucuguc.......................................................................   1        1     Fm1
    ....................uccaggcacugucguucuguA.......................................................................   1        1     Fm1
    ....................uccaggcacugucguucuguU.......................................................................   3        1     Fm1
    ....................uccaggcacugucguucAguc.......................................................................   1        1     Fm1
    ....................uccaggcacugucguucugucU......................................................................   20       1     Fm1
    ....................uccaAgcacugucguucugucg......................................................................   1        1     Fm1
    ....................uUcaggcacugucguucugucg......................................................................   1        1     Fm1
    ....................uccaggcacugucguucugucg......................................................................   2119     0     Fm1
    ....................uccaggcacuUucguucugucg......................................................................   3        1     Fm1
    ....................uccaggcacugucguucGgucg......................................................................   9        1     Fm1
    ....................uccaggcUcugucguucugucg......................................................................   1        1     Fm1
    ....................uccaggcacAgucguucugucg......................................................................   1        1     Fm1
    ....................uccaggcacugucguucugucA......................................................................   5        1     Fm1
    ....................ucGaggcacugucguucugucg......................................................................   2        1     Fm1
    ....................uccaggcacugucguuAugucg......................................................................   3        1     Fm1
    ....................uccaggcacugucguuUugucg......................................................................   5        1     Fm1
    ....................uccaggcacugucguucCgucg......................................................................   17       1     Fm1
    ....................uccaggcacuCucguucugucg......................................................................   2        1     Fm1
    ....................ucUaggcacugucguucugucg......................................................................   5        1     Fm1
    ....................uccagNcacugucguucugucg......................................................................   1        1     Fm1
    ....................uccaUgcacugucguucugucg......................................................................   1        1     Fm1
    ....................uccaggcacugucguucAgucg......................................................................   12       1     Fm1
    ....................uccGggcacugucguucugucg......................................................................   1        1     Fm1
    ....................uccaggcacuAucguucugucg......................................................................   6        1     Fm1
    ....................uccaggcacugucAuucugucg......................................................................   1        1     Fm1
    ....................uccaggcacugucguuGugucg......................................................................   10       1     Fm1
```

Mature Star

```
ucacugacggacucgugggu uccaggcacugucguucugucg cuguaaucguaaugug guagaaugaccuugccugaucc ccaagggaacaucaacaucuggacacuugaac
....................ucAaggcacugucguucugucg......................................................................  1     1  Fm1
....................uccaggcacugucguucugucgc.....................................................................  195   0  Fm1
....................uccaggcacugucguucAgucgc.....................................................................  1     1  Fm1
....................uccaggcacugucguucugucgG.....................................................................  4     1  Fm1
....................uccaggcacugucguucugucgU.....................................................................  51    1  Fm1
....................uccaggcacugucguuAugucgc.....................................................................  1     1  Fm1
....................uccaggcacugucguucugucgA.....................................................................  26    1  Fm1
....................uccaggcacugucguuUugucgc.....................................................................  1     1  Fm1
....................uccaggcacugucguucCgucgc.....................................................................  1     1  Fm1
....................uccaggcacugucguucGgucgc.....................................................................  1     1  Fm1
....................uAcaggcacugucguucugucgc.....................................................................  1     1  Fm1
....................uccaggcacugucguucugucgcA....................................................................  6     1  Fm1
....................uccaggcacugucguucugucgUu....................................................................  5     1  Fm1
....................Nccaggcacugucguucugucgcu....................................................................  1     1  Fm1
....................uccaggcacugucguucugucgcG....................................................................  1     1  Fm1
....................uccaggcacugucguucugucgcu....................................................................  17    0  Fm1
....................uccaggcacugucguucCgucgcu....................................................................  1     1  Fm1
....................uccaggcacugucguucugucgcug...................................................................  1     0  Fm1
....................uccaggcacugucguucugucgcuU...................................................................  6     1  Fm1
.....................ccaggcacugucguucugucgc.....................................................................  5     0  Fm1
......................caggcacugucguucugucg......................................................................  1     0  Fm1
..........................................cuguaaucguaaugugguagaaugaccuugccugaucc................................  1     0  Fm1
...........................................uguaaucguaaugugguagaaugaccuugccugauc.................................  1     0  Fm1
..........................................................guagaaugaccuugccuga...................................  1     0  Fm1
..........................................................guagaaugacGuugccugau..................................  1     1  Fm1
..........................................................guagaaugaccuugccuCau..................................  1     1  Fm1
..........................................................guagaaugaccuugccugau..................................  27    0  Fm1
..........................................................guagaaugaccuugccugUu..................................  1     1  Fm1
..........................................................guagaaugaccuugccugaA..................................  1     1  Fm1
..........................................................guagaaugacAuugccugau..................................  1     1  Fm1
..........................................................guagaaugaccuugccCgauc.................................  2     1  Fm1
..........................................................guagaaugaccuugccugauc.................................  166   0  Fm1
..........................................................guagaaugaccuugccugauU.................................  3     1  Fm1
..........................................................Uuagaaugaccuugccugauc.................................  1     1  Fm1
..........................................................guagaaugaccCugccugauc.................................  1     1  Fm1
..........................................................guagaaugaccuugccuAauc.................................  5     1  Fm1
..........................................................guagaaugaccuugccGgaucc................................  2     1  Fm1
..........................................................guagaaugaccuugccugaAcc................................  2     1  Fm1
..........................................................guagaauUaccuugccugaucc................................  2     1  Fm1
..........................................................guagaaugaccuugccAgaucc................................  9     1  Fm1
..........................................................guagaaugaccuugccugaucc................................  1431  0  Fm1
..........................................................guagaaugacUuugccugaucc................................  2     1  Fm1
..........................................................guagaauAaccuugccugaucc................................  2     1  Fm1
..........................................................guGgaaugaccuugccugaucc................................  1     1  Fm1
..........................................................guagaaugaccuugccuAaucc................................  7     1  Fm1
..........................................................guagaaCgaccuugccugaucc................................  1     1  Fm1
..........................................................guagaaugaccuugccuCaucc................................  2     1  Fm1
..........................................................guagaaugaccuugccugaucA................................  2     1  Fm1
..........................................................guagaaugaccuugccugaCcc................................  1     1  Fm1
..........................................................guagaaugaccuugccCgaucc................................  6     1  Fm1
..........................................................guagaaugaccCugccugaucc................................  2     1  Fm1
..........................................................guagaGugaccuugccugaucc................................  2     1  Fm1
..........................................................guagaaugaccuugccuUaucc................................  2     1  Fm1
..........................................................guagaaugacGuugccugaucc................................  2     1  Fm1
..........................................................guagaaugaccuGgccugaucc................................  1     1  Fm1
..........................................................guagaauCaccuugccugaucc................................  1     1  Fm1
..........................................................guagaaugUccuugccugaucc................................  2     1  Fm1
..........................................................guagaaugaccuugccugaucU................................  39    1  Fm1
..........................................................guagaaugaccuugccugUucc................................  1     1  Fm1
..........................................................guagaaugacAuugccugaucc................................  3     1  Fm1
..........................................................guagGaugaccuugccugaucc................................  1     1  Fm1
..........................................................guagaaugaccuugccugauccA...............................  125   1  Fm1
..........................................................guagaaugaccuugccugauccc...............................  2     0  Fm1
..........................................................guagaaugaccuugccugauccU...............................  508   1  Fm1
..........................................................guagaaugaccuugccugauccG...............................  31    1  Fm1
.............................................................gaaugaccuugccugaucc................................  3     0  Fm1

..................Cuccaggcacugucguucuguc........................................................................  3     1  Fm2
....................uccaggcacugucguucu..........................................................................  2     0  Fm2
....................uccaggcacugucguucug.........................................................................  8     0  Fm2
```

```
                    Mature                                Star
ucacugacggacucgugggauccaggcacugucguucugucgcuguaaucguaaugugguagaaugaccuugccugaucccaagggaacaucaacaucuggacacuugaac
....................uccaggcacugucguucAgu........................................................................  2     1  Fm2
....................uccaggcacugucguucugu........................................................................  26    0  Fm2
....................uccaggcacugucguucugG........................................................................  3     1  Fm2
....................uccaggcacugucguucuUuc.......................................................................  1     1  Fm2
....................uccaggcacugucguucuguA.......................................................................  10    1  Fm2
....................uccaggcacugucguucuguc.......................................................................  121   0  Fm2
....................uccaggcacugucguucuguU.......................................................................  10    1  Fm2
....................uccagUcacugucguucugucg......................................................................  1     1  Fm2
....................uccaggcacugucAuucugucg......................................................................  1     1  Fm2
....................uccaggcacugucguucGgucg......................................................................  5     1  Fm2
....................uccaggcacugucguucuguAg......................................................................  1     1  Fm2
....................uccaggcacugucguucugucg......................................................................  1102  0  Fm2
....................uccaggcacugucguucuUucg......................................................................  1     1  Fm2
....................uccaggcacugucguuGugucg......................................................................  2     1  Fm2
....................uccaggcacugucguucCgucg......................................................................  6     1  Fm2
....................Nccaggcacugucguucugucg......................................................................  2     1  Fm2
....................ucAaggcacugucguucugucg......................................................................  1     1  Fm2
....................uGcaggcacugucguucugucg......................................................................  1     1  Fm2
....................uccaggcacugucguucugucA......................................................................  6     1  Fm2
....................ucUaggcacugucguucugucg......................................................................  1     1  Fm2
....................uccaggcacugucguuUugucg......................................................................  1     1  Fm2
....................ucGaggcacugucguucugucg......................................................................  1     1  Fm2
....................uccaggcacuUucguucugucg......................................................................  2     1  Fm2
....................uccaggcUcugucguucugucg......................................................................  1     1  Fm2
....................uccGggcacugucguucugucg......................................................................  1     1  Fm2
....................uccaggcacugucguucugGcg......................................................................  1     1  Fm2
....................uccaggcacugucgCucugucg......................................................................  1     1  Fm2
....................uccaggcacuAucguucugucg......................................................................  2     1  Fm2
....................uccaggcacugucguucAgucg......................................................................  8     1  Fm2
....................uUcaggcacugucguucugucg......................................................................  1     1  Fm2
....................uccaggcacugucguucugucU......................................................................  2     1  Fm2
....................uccaggcacugucguuAugucg......................................................................  3     1  Fm2
....................uccaggcacugucguucugucgU.....................................................................  31    1  Fm2
....................uccaggcacugucguucugucgA.....................................................................  83    1  Fm2
....................uccaggcacugucguucAgucgc.....................................................................  1     1  Fm2
....................uccaggcacugucguucCgucgc.....................................................................  1     1  Fm2
....................uccaggcacugucguucugucgc.....................................................................  117   0  Fm2
....................uccaggcacugucguucGgucgc.....................................................................  1     1  Fm2
....................uccaggcacugucguucugucgG.....................................................................  2     1  Fm2
....................uccaggcacugucguucugucgcu....................................................................  91    0  Fm2
....................uccaggcacugucguucugucgcA....................................................................  9     1  Fm2
....................uccaggcacugucguucugucgcG....................................................................  7     1  Fm2
....................uccaggcacugucguucugucgUu....................................................................  13    1  Fm2
....................uccaggcacugucguucugucgcuU...................................................................  18    1  Fm2
......................caggcacugucguucugucg......................................................................  9     0  Fm2
......................caggcacugucguAcugucg......................................................................  1     1  Fm2
..........................................................guagaaugaccuugccugau..................................  5     0  Fm2
..........................................................guagaaugaccuugccuAauc.................................  2     1  Fm2
..........................................................Nuagaaugaccuugccugauc.................................  1     1  Fm2
..........................................................guagaaugaccuugcAugauc.................................  1     1  Fm2
..........................................................guagaaugacAuugccugauc.................................  1     1  Fm2
..........................................................guagaaugaccuugccugauc.................................  177   0  Fm2
..........................................................guagaaugaccuugccugCuc.................................  1     1  Fm2
..........................................................guagaaugaccuugccugauU.................................  2     1  Fm2
..........................................................guagaaugaccuugccugaAcc................................  2     1  Fm2
..........................................................guagUaugaccuugccugaucc................................  1     1  Fm2
..........................................................guagGaugaccuugccugaucc................................  1     1  Fm2
..........................................................guagaaugaccuugccuUaucc................................  7     1  Fm2
..........................................................guagaaCgaccuugccugaucc................................  1     1  Fm2
..........................................................guagaaugaAcuugccugaucc................................  2     1  Fm2
..........................................................guagaaugacAuugccugaucc................................  3     1  Fm2
..........................................................guagaaugaccuugccugaucc................................  1296  0  Fm2
..........................................................guagaauCaccuugccugaucc................................  1     1  Fm2
..........................................................guagaaugaccuugAcugaucc................................  1     1  Fm2
..........................................................guagaaugaccuugUcugaucc................................  1     1  Fm2
..........................................................guagaaugacGuugccugaucc................................  2     1  Fm2
..........................................................guagaaugaccuugccuAaucc................................  3     1  Fm2
..........................................................guagaaugaccuugccugaucU................................  27    1  Fm2
..........................................................guagaaugaccuugccugauAc................................  1     1  Fm2
..........................................................guagaaugaccuugccCgaucc................................  3     1  Fm2
```

```
                    Mature                                  Star
ucacugacggacucgugggu uccaggcacugucguucugucg cuguaaucguaaugug guagaaugaccuugccugaucc ccaagggaacaucaacaucuggacacuugaac
```

```
ucacugacggacucguggguuccaggcacugucguucugucgcuguaaucguaaugugguagaaugaccuugccugauccccaagggaacaucaacaucuggacacuugaac
..........................................................guUgaaugaccuugccugaucc................................  1     1  Fm2
..........................................................guagaUugaccuugccugaucc................................  1     1  Fm2
..........................................................guagaaugaccuugccugUucc................................  1     1  Fm2
..........................................................guagaaugGccuugccugaucc................................  1     1  Fm2
..........................................................guagaaugacUuugccugaucc................................  3     1  Fm2
..........................................................guagaaugaccuugccuCaucc................................  6     1  Fm2
..........................................................guagaaugaccuugccGgaucc................................  2     1  Fm2
..........................................................guagaaugaccuugccAgaucc................................  2     1  Fm2
..........................................................guagaauAaccuugccugaucc................................  1     1  Fm2
..........................................................guagaaugaccuugccugauccG...............................  9     1  Fm2
..........................................................guagaaugaccuugccugauccA...............................  45    1  Fm2
..........................................................guagaaugaccuugccugauccU...............................  114   1  Fm2

....................uccaggcacugucguuUu..........................................................................  1     1  Mm2
....................uccaggcacugucguucA..........................................................................  1     1  Mm2
....................uccaggcacugucguuAu..........................................................................  1     1  Mm2
....................uccaggcacugucguucu..........................................................................  24    0  Mm2
....................uccaggcacugucguucuC.........................................................................  1     1  Mm2
....................uccaggcacugucguucuU.........................................................................  5     1  Mm2
....................uccaggcacugucguucug.........................................................................  27    0  Mm2
....................uccaggcacugucguucugu........................................................................  129   0  Mm2
....................uccaggcacugucguucCgu........................................................................  2     1  Mm2
....................uccaggcacugucguucAgu........................................................................  1     1  Mm2
....................uccaggcacugucguucGgu........................................................................  1     1  Mm2
....................uccaggcacugucguucuguU.......................................................................  4     1  Mm2
....................uccaggcacuUucguucuguc.......................................................................  1     1  Mm2
....................uccaggcacugucguucAguc.......................................................................  5     1  Mm2
....................uccaggcacugucguuAuguc.......................................................................  1     1  Mm2
....................uccaggcacugucguucuguA.......................................................................  6     1  Mm2
....................uccaggcacuAucguucuguc.......................................................................  1     1  Mm2
....................uccaggcaUugucguucuguc.......................................................................  1     1  Mm2
....................uccaggcacugucguucGguc.......................................................................  4     1  Mm2
....................uccaggcacugucguucuguG.......................................................................  1     1  Mm2
....................uccaggcacugucguucCguc.......................................................................  3     1  Mm2
....................uccaggUacugucguucuguc.......................................................................  1     1  Mm2
....................uccaggcacugucguucuguc.......................................................................  564   0  Mm2
....................Cccaggcacugucguucuguc.......................................................................  1     1  Mm2
....................uccaggcacugucguucugucC......................................................................  1     1  Mm2
....................uccaggcacugucUuucugucg......................................................................  1     1  Mm2
....................uccaggcacugucguucCgucg......................................................................  37    1  Mm2
....................uccaggcacugucguucuguAg......................................................................  3     1  Mm2
....................uccaggcacGgucguucugucg......................................................................  1     1  Mm2
....................uccagUcacugucguucugucg......................................................................  5     1  Mm2
....................uccaggcacugucAuucugucg......................................................................  2     1  Mm2
....................uccaggcacuAucguucugucg......................................................................  9     1  Mm2
....................uAcaggcacugucguucugucg......................................................................  3     1  Mm2
....................uccaggcacuCucguucugucg......................................................................  7     1  Mm2
....................uGcaggcacugucguucugucg......................................................................  3     1  Mm2
....................uccaggcacugucguucugucg......................................................................  6199  0  Mm2
....................uccaggcUcugucguucugucg......................................................................  3     1  Mm2
....................uccaggcacuUucguucugucg......................................................................  4     1  Mm2
....................uccaggcGcugucguucugucg......................................................................  4     1  Mm2
....................uccaggcacugucguucugAcg......................................................................  2     1  Mm2
....................Gccaggcacugucguucugucg......................................................................  1     1  Mm2
....................Cccaggcacugucguucugucg......................................................................  2     1  Mm2
....................uccaggUacugucguucugucg......................................................................  3     1  Mm2
....................uccaggcacuguAguucugucg......................................................................  1     1  Mm2
....................uccGggcacugucguucugucg......................................................................  3     1  Mm2
....................uccaggcacugucguucuguUg......................................................................  2     1  Mm2
....................ucAaggcacugucguucugucg......................................................................  1     1  Mm2
....................uccaggcaUugucguucugucg......................................................................  3     1  Mm2
....................ucUaggcacugucguucugucg......................................................................  2     1  Mm2
....................Nccaggcacugucguucugucg......................................................................  1     1  Mm2
....................uccaggcacugucguucAgucg......................................................................  35    1  Mm2
....................uccaggcacugucguucugCcg......................................................................  1     1  Mm2
....................uccaggcacuguUguucugucg......................................................................  1     1  Mm2
....................uccaggcacugucguucuAucg......................................................................  2     1  Mm2
....................uccaggcacugucgAucugucg......................................................................  1     1  Mm2
....................uccaggcacugucguucugucU......................................................................  105   1  Mm2
....................uccaggcaGugucguucugucg......................................................................  1     1  Mm2
```

Mature **Star**

ucacugacggacucgugggu**uccaggcacugucguucugucg**cuguaaucguaaugug**guagaaugaccuugccugaucc**ccaagggaacaucaacaucuggacacuugaac			
....................uccaggcacugucguuAugucg..	38	1	Mm2
....................uccaggcacugucguucGgucg..	27	1	Mm2
....................uccagCcacugucguucugucg..	1	1	Mm2
....................uccaggcCcugucguucugucg..	1	1	Mm2
....................ucGaggcacugucguucugucg..	2	1	Mm2
....................uccaggcacugucguuUugucg..	6	1	Mm2
....................uccaggcacugucguucugucA..	14	1	Mm2
....................uccaggcacugucguuGugucg..	9	1	Mm2
....................uccaggcacugucguucCgucgc...	4	1	Mm2
....................uccUggcacugucguucugucgc...	1	1	Mm2
....................uccaggcacuCucguucugucgc...	2	1	Mm2
....................uccaggcacugucguucugucgU...	291	1	Mm2
....................uccaggcacugucguucAgucgc...	1	1	Mm2
....................uccaggcacugucguuGugucgc...	1	1	Mm2
....................uccaggcacugucguuUugucgc...	1	1	Mm2
....................uccaggcacugucguucugucgG...	9	1	Mm2
....................uccaggcacugucguucugucgA...	145	1	Mm2
....................uccaggcacugucguucugucUc...	1	1	Mm2
....................uccaggcacugucguuAugucgc...	3	1	Mm2
....................uccaggcacugucguucugCcgc...	1	1	Mm2
....................uccaggcacugucguucugucgc...	570	0	Mm2
....................uccaggcacugucguucGgucgc...	1	1	Mm2
....................uccaggcacugucguucAgucgcu..	1	1	Mm2
....................uccaggcacugucguucugucgUu..	78	1	Mm2
....................uccaggcacugucguucugucgAu..	1	1	Mm2
....................uccaggcacuguUguucugucgcu..	1	1	Mm2
....................uccaggcacugucguucugucgcu..	120	0	Mm2
....................ucGaggcacugucguucugucgcu..	1	1	Mm2
....................uccaggcacugucguucugucgcG..	9	1	Mm2
....................uccaggcacugucguucugucgcA..	24	1	Mm2
....................uccaggcacugucguucugucgcuU...	22	1	Mm2
....................uccaggcacugucguucugucgcuguU...	1	1	Mm2
....................uccaggcacugucguucugucgcuguaau...	1	0	Mm2
.....................ccaggcacugucguucugucg..	2	0	Mm2
.....................Ucaggcacugucguucugucg..	1	1	Mm2
.....................ccaggcacugucguucugucgc...	2	0	Mm2
......................caggcacugucguucuguc...	2	0	Mm2
......................caggcacugucguucugAcg..	2	1	Mm2
......................caggAacugucguucugucg..	1	1	Mm2
......................caggcacugucguucugucg..	21	0	Mm2
......................caggcacugucguucugucgc...	1	0	Mm2
......................caggcacugucguucugucgcA..	1	1	Mm2
.......................aggcacugucguucuguAg..	1	1	Mm2
.......................aggcacugucguucugucg..	1	0	Mm2
....cgcuguaaucguaaugugguagaaugaccuugccu.....................................	1	0	Mm2
....cuguaaucguaaugugguagaaugacc...	2	0	Mm2
....cuguaaucguaaugugguagaaugaccuu...	1	0	Mm2
....uguaaucguaaugugguagaaug..	1	0	Mm2
....uguaaucguaaugugguagaaugacc...	1	0	Mm2
....uguaaucguaaugugguagaaugaccuug..	2	0	Mm2
....uguaaucguaaugugguagaaugaccuugccu.....................................	1	0	Mm2
....uguaaucguaaugugguagaaugaccuugccugauc.................................	1	0	Mm2
....gguagaaugaccuugccugaucc................................	1	0	Mm2
....guagaaugaccuugccuga...................................	14	0	Mm2
....guagaaugaccuugccugU...................................	1	1	Mm2
....guagaaugaccuugccugau..................................	44	0	Mm2
....guagaaugaUcuugccugau..................................	1	1	Mm2
....guagaaugaccuugAcugauc.................................	1	1	Mm2
....guagaaugaccuugccugauc.................................	261	0	Mm2
....guagaaugaccuugccuAauc.................................	1	1	Mm2
....guagaaugaccuugccugauU.................................	7	1	Mm2
....guaCaaugaccuugccugauc.................................	1	1	Mm2
....Uuagaaugaccuugccugauc.................................	1	1	Mm2
....guagaaugaccuugccuCauc.................................	1	1	Mm2
....guagaaugaccuugccuUauc.................................	2	1	Mm2
....guagaaugUccuugccugauc.................................	1	1	Mm2
....guagaaugaccuugccugauA.................................	1	1	Mm2
....guagaaugaccuugccugaucU................................	44	1	Mm2
....Auagaaugaccuugccugaucc................................	1	1	Mm2
....guagaaugacGuugccugaucc................................	5	1	Mm2

```
                    Mature                                    Star
ucacugacggacucguggguuccaggcacugucguucugucgcuguaaucguaaugugguagaaugaccuugccugaucccaagggaacaucaacaucuggacacuugaac
..........................................................guagUaugaccuugccugaucc................................  1     1  Mm2
..........................................................guagaaugaccuugccugauAc................................  1     1  Mm2
..........................................................guagaauUaccuugccugaucc................................  1     1  Mm2
..........................................................guagaaugGccuugccugaucc................................  2     1  Mm2
..........................................................guaUaaugaccuugccugaucc................................  2     1  Mm2
..........................................................guagaaugaccuugccGgaucc................................  9     1  Mm2
..........................................................guagaaugaccuugccugaAcc................................  1     1  Mm2
..........................................................guagaaAgaccuugccugaucc................................  2     1  Mm2
..........................................................guagaaugCccuugccugaucc................................  6     1  Mm2
..........................................................guagaauCaccuugccugaucc................................  1     1  Mm2
..........................................................guagaaugaccuugccuCaucc................................  12    1  Mm2
..........................................................guaAaaugaccuugccugaucc................................  1     1  Mm2
..........................................................guagaaugUccuugccugaucc................................  2     1  Mm2
..........................................................guagCaugaccuugccugaucc................................  1     1  Mm2
..........................................................guagaaugacUuugccugaucc................................  2     1  Mm2
..........................................................guagaaugaccuugccugaucc................................  3318  0  Mm2
..........................................................guagaaugaccuugccCgaucc................................  8     1  Mm2
..........................................................guagaaugaccuugccugaucA................................  2     1  Mm2
..........................................................guGgaaugaccuugccugaucc................................  1     1  Mm2
..........................................................guagGaugaccuugccugaucc................................  1     1  Mm2
..........................................................guagaaugacAuugccugaucc................................  12    1  Mm2
..........................................................guagaaugaccAugccugaucc................................  1     1  Mm2
..........................................................guagaaugaccuugccAgaucc................................  9     1  Mm2
..........................................................guagaaugaccuugccugGucc................................  1     1  Mm2
..........................................................guagaaugaccuuUccugaucc................................  2     1  Mm2
..........................................................Uuagaaugaccuugccugaucc................................  1     1  Mm2
..........................................................guagaaugaccuuAccugaucc................................  1     1  Mm2
..........................................................guagaaugaccuugccuAaucc................................  16    1  Mm2
..........................................................guagaaugaccuugccugauUc................................  1     1  Mm2
..........................................................guagaaugaccuugccuUaucc................................  18    1  Mm2
..........................................................guagaaugaccuugAcugaucc................................  1     1  Mm2
..........................................................guagaaugaccCugccugaucc................................  1     1  Mm2
..........................................................guagaGugaccuugccugaucc................................  1     1  Mm2
..........................................................guagaaugaccuugccugauccU...............................  540   1  Mm2
..........................................................guagaaugaccuugccugauccG...............................  19    1  Mm2
..........................................................guagaaugaccuugccugauccA...............................  114   1  Mm2
..........................................................guagaaugaccuugccugauccc...............................  1     0  Mm2
..........................................................guagaaugaccuugccugauccUc..............................  2     1  Mm2
...........................................................uagaaugaccuugccugaucc................................  2     0  Mm2

...................Cuccaggcacugucguucugucg......................................................................  8     1  Mm1
....................uccaggcacugucguucu..........................................................................  5     0  Mm1
....................uccaggcacugucguucug.........................................................................  16    0  Mm1
....................uccaggcacugucguucCgu........................................................................  2     1  Mm1
....................uccaggcacugucguucugG........................................................................  1     1  Mm1
....................uccaggcacugucguucGgu........................................................................  1     1  Mm1
....................uccaggcacugucguucAgu........................................................................  2     1  Mm1
....................uccaggcacugucguucugu........................................................................  152   0  Mm1
....................uccaggcacugucguucAguc.......................................................................  4     1  Mm1
....................uccaggcacugucguuAuguc.......................................................................  2     1  Mm1
....................uccaggcacugucguucuguc.......................................................................  479   0  Mm1
....................uccaggcacugucguCcuguc.......................................................................  1     1  Mm1
....................uccaggcacugucguucGguc.......................................................................  2     1  Mm1
....................uccaggcacugucguucCguc.......................................................................  3     1  Mm1
....................uccaggcacugucguuAugucg......................................................................  29    1  Mm1
....................uccaggcacugucguucugucU......................................................................  86    1  Mm1
....................uUcaggcacugucguucugucg......................................................................  18    1  Mm1
....................uccGggcacugucguucugucg......................................................................  1     1  Mm1
....................uccaggcacuUucguucugucg......................................................................  4     1  Mm1
....................uccaggcacugucgAucugucg......................................................................  1     1  Mm1
....................uccaggcUcugucguucugucg......................................................................  2     1  Mm1
....................ucAaggcacugucguucugucg......................................................................  2     1  Mm1
....................Cccaggcacugucguucugucg......................................................................  2     1  Mm1
....................Accaggcacugucguucugucg......................................................................  2     1  Mm1
....................uccaggcacugucguucuguUg......................................................................  1     1  Mm1
....................uccaggcacugucAuucugucg......................................................................  1     1  Mm1
....................uccaggcacugucguuGugucg......................................................................  12    1  Mm1
....................uccaggcacugucguucuguAg......................................................................  1     1  Mm1
....................uccaggcacugucguucAgucg......................................................................  43    1  Mm1
....................uccaggcacuCucguucugucg......................................................................  5     1  Mm1
```

```
                    Mature                                   Star
ucacugacggacucgugggu uccaggcacugucguucugucg cuguaaucguaaugug guagaaugaccuugccugaucc ccaagggaacaucaacaucuggacacuugaac
....................uccaggcacugucguucugucg......................................................................    6904   0    Mm1
....................uccaggcacugucguucugAcg......................................................................    2      1    Mm1
....................uccagUcacugucguucugucg......................................................................    3      1    Mm1
....................uccaggcacuguUguucugucg......................................................................    1      1    Mm1
....................uccaggcacugucgCucugucg......................................................................    2      1    Mm1
....................uccaggcacugucguAcugucg......................................................................    1      1    Mm1
....................Nccaggcacugucguucugucg......................................................................    3      1    Mm1
....................uAcaggcacugucguucugucg......................................................................    4      1    Mm1
....................uccaggcacuAucguucugucg......................................................................    15     1    Mm1
....................ucGaggcacugucguucugucg......................................................................    1      1    Mm1
....................uccaggcacugucguuUugucg......................................................................    11     1    Mm1
....................uccaggcacugucguucuCucg......................................................................    1      1    Mm1
....................uccaggcacugCcguucugucg......................................................................    2      1    Mm1
....................uccaAgcacugucguucugucg......................................................................    1      1    Mm1
....................uccaggcacugucguucugucA......................................................................    9      1    Mm1
....................uccaggcacugucguucGgucg......................................................................    36     1    Mm1
....................uccaCgcacugucguucugucg......................................................................    1      1    Mm1
....................uccagNcacugucguucugucg......................................................................    2      1    Mm1
....................uccaUgcacugucguucugucg......................................................................    1      1    Mm1
....................uccaggcacugucguucuAucg......................................................................    2      1    Mm1
....................uccaggcGcugucguucugucg......................................................................    1      1    Mm1
....................ucUaggcacugucguucugucg......................................................................    1      1    Mm1
....................uccaggcacugucguucCgucg......................................................................    36     1    Mm1
....................uccUggcacugucguucugucg......................................................................    3      1    Mm1
....................uccaggcacAgucguucugucg......................................................................    1      1    Mm1
....................uccaggcacuCucguucugucgc.....................................................................    1      1    Mm1
....................uccaggcacugucguucugucgU.....................................................................    413    1    Mm1
....................uccaggcacugucguucugucgA.....................................................................    252    1    Mm1
....................uccaggcacugucguucAgucgc.....................................................................    5      1    Mm1
....................uccaggcacugucguucCgucgc.....................................................................    2      1    Mm1
....................uccaggcacugucguuGugucgc.....................................................................    2      1    Mm1
....................uccaggcacugucguucGgucgc.....................................................................    2      1    Mm1
....................uccagAcacugucguucugucgc.....................................................................    1      1    Mm1
....................Gccaggcacugucguucugucgc.....................................................................    1      1    Mm1
....................uccaggcacugucguucuguAgc.....................................................................    1      1    Mm1
....................uccaggcacugucguuAugucgc.....................................................................    1      1    Mm1
....................uccaggcacugucguucugucgc.....................................................................    470    0    Mm1
....................uccaggcacugucguucugucgcA....................................................................    69     1    Mm1
....................uccaggcacugucguucugucgAu....................................................................    11     1    Mm1
....................uccaggcacugucguucugucgUu....................................................................    140    1    Mm1
....................uccaggcacugucguucugucgcu....................................................................    81     0    Mm1
....................Nccaggcacugucguucugucgcu....................................................................    2      1    Mm1
....................uccaggcacugucguucugucgcuU...................................................................    34     1    Mm1
....................uccaggcacugucguucugucgcuA...................................................................    1      1    Mm1
....................uccaggcacugucguucugucgcuguaaucguaauguggu....................................................    1      0    Mm1
......................caggcacugucguucugCcg......................................................................    1      1    Mm1
......................caggcacugucguucuUucg......................................................................    1      1    Mm1
......................caggcacugucguucugucg......................................................................    14     0    Mm1
...............................................aucguaaugugguagaaugaccuugccugauc.................................    1      0    Mm1
..........................................................guagaaugaccuugccugau..................................    3      0    Mm1
..........................................................guagaaugaccuugccuUauc.................................    1      1    Mm1
..........................................................guagaaugaccuugccugauc.................................    33     0    Mm1
..........................................................guagaaugaccuugccAgaucc................................    1      1    Mm1
..........................................................guagaaugaccuugccuUaucc................................    3      1    Mm1
..........................................................guagaaugacGuugccugaucc................................    1      1    Mm1
..........................................................guagaaugaccuugccugaucA................................    1      1    Mm1
..........................................................guagaaugaccuugccugaucc................................    200    0    Mm1
..........................................................guagUaugaccuugccugaucc................................    1      1    Mm1
..........................................................guagaaugaccuCgccugaucc................................    1      1    Mm1
..........................................................guagaaugacAuugccugaucc................................    1      1    Mm1
..........................................................guagaaugaccuugccugauccU...............................    112    1    Mm1
...........................................................uagaaugaccuugccugaucc................................    5      0    Mm1

...................Cuccaggcacugucguucugucg......................................................................    8      1    Fm3
....................uccaggcacugucAuucu..........................................................................    1      1    Fm3
....................uccaggcacugucguucu..........................................................................    63     0    Fm3
....................uccaggcacugucguuAu..........................................................................    6      1    Fm3
....................uccaggcacugucguucuU.........................................................................    2      1    Fm3
....................uccaggcacugucguucug.........................................................................    66     0    Fm3
....................uccaggcacugucguuAug.........................................................................    2      1    Fm3
```

```
                    Mature                                    Star
ucacugacggacucgugggguuccaggcacugucguucugucgcuguaaucguaaugugguagaaugaccuugccugauccccaagggaacaucaacaucuggacacuugaac
....................uccaggcacugucguucGgu........................................................................ 3      1   Fm3
....................ucAaggcacugucguucugu........................................................................ 1      1   Fm3
....................uccaggcacugucguuUugu........................................................................ 2      1   Fm3
....................uccaggcacuAucguucugu........................................................................ 1      1   Fm3
....................uccaggcacugucguucugA........................................................................ 6      1   Fm3
....................uccaggcacuCucguucugu........................................................................ 2      1   Fm3
....................uccaggcacugucguucCgu........................................................................ 3      1   Fm3
....................uccaggcacugucguuGugu........................................................................ 1      1   Fm3
....................Accaggcacugucguucugu........................................................................ 1      1   Fm3
....................uccaggcacugucguucugu........................................................................ 636    0   Fm3
....................uccaggcacugucguucAgu........................................................................ 6      1   Fm3
....................uccaggcacugucguuAugu........................................................................ 3      1   Fm3
....................uAcaggcacugucguucugu........................................................................ 1      1   Fm3
....................Nccaggcacugucguucuguc....................................................................... 2      1   Fm3
....................uccaggcacugucguuUuguc....................................................................... 4      1   Fm3
....................uccaggcacugucguucuAuc....................................................................... 2      1   Fm3
....................uccaUgcacugucguucuguc....................................................................... 1      1   Fm3
....................ucAaggcacugucguucuguc....................................................................... 3      1   Fm3
....................Accaggcacugucguucuguc....................................................................... 1      1   Fm3
....................ucGaggcacugucguucuguc....................................................................... 1      1   Fm3
....................uAcaggcacugucguucuguc....................................................................... 2      1   Fm3
....................uccaggcacuAucguucuguc....................................................................... 2      1   Fm3
....................uccaggcacuUucguucuguc....................................................................... 1      1   Fm3
....................uccaggcacugucguucuguc....................................................................... 2107   0   Fm3
....................uccaggcacugucguucAguc....................................................................... 14     1   Fm3
....................uccaggcacugucguuAuguc....................................................................... 7      1   Fm3
....................uccaggcGcugucguucuguc....................................................................... 1      1   Fm3
....................uccaggcaUugucguucuguc....................................................................... 10     1   Fm3
....................uccaggcacugGcguucuguc....................................................................... 1      1   Fm3
....................uccaggcacugucguucuguG....................................................................... 1      1   Fm3
....................uccaggcacugucguucuguU....................................................................... 47     1   Fm3
....................uccGggcacugucguucuguc....................................................................... 2      1   Fm3
....................ucUaggcacugucguucuguc....................................................................... 1      1   Fm3
....................uccaggcacugucguuGuguc....................................................................... 3      1   Fm3
....................uccaggcacugucguucuguA....................................................................... 42     1   Fm3
....................uccaggcacuguUguucuguc....................................................................... 1      1   Fm3
....................uccaggcacuCucguucuguc....................................................................... 4      1   Fm3
....................uccaggcacugucguucCguc....................................................................... 16     1   Fm3
....................uccaggcacugucguucGguc....................................................................... 8      1   Fm3
....................uccaggcacugucgAucuguc....................................................................... 1      1   Fm3
....................uUcaggcacugucguucuguc....................................................................... 1      1   Fm3
....................uccaAgcacugucguucuguc....................................................................... 1      1   Fm3
....................uccaggcacugucguAcugucg...................................................................... 5      1   Fm3
....................uccCggcacugucguucugucg...................................................................... 2      1   Fm3
....................uccaggcaUugucguucugucg...................................................................... 9      1   Fm3
....................uccaggAacugucguucugucg...................................................................... 7      1   Fm3
....................uccaggcacugucguucuguUg...................................................................... 7      1   Fm3
....................uccaAgcacugucguucugucg...................................................................... 5      1   Fm3
....................uccaggcacugucgCucugucg...................................................................... 5      1   Fm3
....................uccagCcacugucguucugucg...................................................................... 8      1   Fm3
....................uccaggcacugucguucugucU...................................................................... 432    1   Fm3
....................uccaCgcacugucguucugucg...................................................................... 2      1   Fm3
....................uccaggcacugucguuGugucg...................................................................... 37     1   Fm3
....................uccaggcacugCcguucugucg...................................................................... 5      1   Fm3
....................Gccaggcacugucguucugucg...................................................................... 1      1   Fm3
....................uccaggcacuguAguucugucg...................................................................... 7      1   Fm3
....................ucUaggcacugucguucugucg...................................................................... 12     1   Fm3
....................uccaggcacugucguucCgucg...................................................................... 169    1   Fm3
....................uAcaggcacugucguucugucg...................................................................... 22     1   Fm3
....................uccaggcacugucguucugucA...................................................................... 71     1   Fm3
....................uccaggcacugucguucugucg...................................................................... 32238  0   Fm3
....................uccaggcacugucguucGgucg...................................................................... 158    1   Fm3
....................uccaggcacuAucguucugucg...................................................................... 57     1   Fm3
....................uccagNcacugucguucugucg...................................................................... 5      1   Fm3
....................uccaggcacugucguucuUucg...................................................................... 9      1   Fm3
....................uccaggcacugucguucuguAg...................................................................... 2      1   Fm3
....................uccaggcacugucguuAugucg...................................................................... 152    1   Fm3
....................uccaggcacugAcguucugucg...................................................................... 3      1   Fm3
....................uccaggNacugucguucugucg...................................................................... 2      1   Fm3
....................uccaUgcacugucguucugucg...................................................................... 5      1   Fm3
```

```
                    Mature                                      Star
ucacugacggacucgugggucccaggcacugucguucugucgcuguaaucguaaugugguagaaugaccuugccugaucccaagggaacaucaacaucuggacacuugaac
....................uUcaggcacugucguucugucg......................................................................   3      1   Fm3
....................uccaggcacugucguuUugucg......................................................................   61     1   Fm3
....................uccaggUacugucguucugucg......................................................................   6      1   Fm3
....................ucAaggcacugucguucugucg......................................................................   19     1   Fm3
....................uccaggcacugucguucugucC......................................................................   4      1   Fm3
....................uccaggcacGgucguucugucg......................................................................   3      1   Fm3
....................uccaggcacugucguucugCcg......................................................................   2      1   Fm3
....................uccaggcacugucguucAgucg......................................................................   166    1   Fm3
....................uccaggcaAugucguucugucg......................................................................   1      1   Fm3
....................uccaggGacugucguucugucg......................................................................   3      1   Fm3
....................uccaggcacCgucguucugucg......................................................................   4      1   Fm3
....................Cccaggcacugucguucugucg......................................................................   9      1   Fm3
....................uccagUcacugucguucugucg......................................................................   3      1   Fm3
....................uccaggcacugucguCcugucg......................................................................   7      1   Fm3
....................uccGggcacugucguucugucg......................................................................   3      1   Fm3
....................uccaggcacugucguucuCucg......................................................................   2      1   Fm3
....................uccaggcacugucguucuAucg......................................................................   13     1   Fm3
....................uccaggcacuUucguucugucg......................................................................   20     1   Fm3
....................uccaggcacugucCuucugucg......................................................................   2      1   Fm3
....................uccaggcacugucguGcugucg......................................................................   2      1   Fm3
....................uccaggcacugucgGucugucg......................................................................   1      1   Fm3
....................uccagAcacugucguucugucg......................................................................   1      1   Fm3
....................uccaggcacugucUuucugucg......................................................................   5      1   Fm3
....................uccaggcacuCucguucugucg......................................................................   28     1   Fm3
....................uGcaggcacugucguucugucg......................................................................   8      1   Fm3
....................uccaggcacugucAuucugucg......................................................................   17     1   Fm3
....................uccaggcacuguUguucugucg......................................................................   12     1   Fm3
....................uccUggcacugucguucugucg......................................................................   2      1   Fm3
....................uccaggcUcugucguucugucg......................................................................   7      1   Fm3
....................uccaggcacugucgAucugucg......................................................................   2      1   Fm3
....................Nccaggcacugucguucugucg......................................................................   17     1   Fm3
....................Accaggcacugucguucugucg......................................................................   6      1   Fm3
....................uccaggcGcugucguucugucg......................................................................   2      1   Fm3
....................uccaggcacugucguucugAcg......................................................................   3      1   Fm3
....................uccaggcacugucguucuguGg......................................................................   2      1   Fm3
....................ucGaggcacugucguucugucg......................................................................   18     1   Fm3
....................uccaggcacuguGguucugucg......................................................................   2      1   Fm3
....................uccaggcacugucguucGgucgc.....................................................................   7      1   Fm3
....................uccaggcacugucguuAugucgc.....................................................................   10     1   Fm3
....................ucAaggcacugucguucugucgc.....................................................................   2      1   Fm3
....................uccaggcacugucguucugucgc.....................................................................   1871   0   Fm3
....................uccaggcaUugucguucugucgc.....................................................................   3      1   Fm3
....................uccaggcacuAucguucugucgc.....................................................................   8      1   Fm3
....................uccaUgcacugucguucugucgc.....................................................................   1      1   Fm3
....................Cccaggcacugucguucugucgc.....................................................................   1      1   Fm3
....................uccUggcacugucguucugucgc.....................................................................   2      1   Fm3
....................Accaggcacugucguucugucgc.....................................................................   1      1   Fm3
....................uccaggcacugucAuucugucgc.....................................................................   2      1   Fm3
....................uccaggcacGgucguucugucgc.....................................................................   1      1   Fm3
....................uccaggcacugucguucuguUgc.....................................................................   1      1   Fm3
....................uccaggcacugucguucugucgG.....................................................................   4      1   Fm3
....................uccaggcacugucguucugucUc.....................................................................   1      1   Fm3
....................uccaggcacugucguucCgucgc.....................................................................   16     1   Fm3
....................uccaggcacugucguucugucgA.....................................................................   1521   1   Fm3
....................uccaggcacugucguucugucgU.....................................................................   897    1   Fm3
....................uccGggcacugucguucugucgc.....................................................................   1      1   Fm3
....................uccaggcacugucguucAgucgc.....................................................................   11     1   Fm3
....................uccaggcacugucguuUugucgc.....................................................................   1      1   Fm3
....................uccaggcacugucguucugucAc.....................................................................   1      1   Fm3
....................Nccaggcacugucguucugucgc.....................................................................   2      1   Fm3
....................ucUaggcacugucguucugucgc.....................................................................   1      1   Fm3
....................uccaggcacuCucguucugucgc.....................................................................   1      1   Fm3
....................uccaggcacuUucguucugucgc.....................................................................   1      1   Fm3
....................uccagUcacugucguucugucgc.....................................................................   1      1   Fm3
....................uccaggcacuguGguucugucgc.....................................................................   1      1   Fm3
....................uccaggcacuguAguucugucgc.....................................................................   1      1   Fm3
....................uccaggcacugucguuGugucgc.....................................................................   1      1   Fm3
....................uccaggcacugucguucugucgcA....................................................................   168    1   Fm3
....................uccaggcacugucguucugucgGu....................................................................   5      1   Fm3
....................uccaggcacugucguucGgucgcu....................................................................   2      1   Fm3
```

Mature **Star**

ucacugacggacucgugggu**uccaggcacugucguucugucg**cuguaaucguaaugug**guagaaugaccuugccugaucc**ccaagggaacaucaacaucuggacacuugaac			
....................uccaggcacugucguucugucgAu..	8	1	Fm3
....................uccaggcacugucguucugucgcG..	6	1	Fm3
....................uccaggcacugucguuUugucgcu..	2	1	Fm3
....................uccaggcacugucguucugucgcu..	251	0	Fm3
....................uccaggcacugucguucugucgUu..	341	1	Fm3
....................uccaggcacugucguucCgucgcu..	3	1	Fm3
....................uccaggcacugucguucAgucgcu..	1	1	Fm3
....................uccaggcacugucguucugucgAug...	7	1	Fm3
....................uccaggcacugucguucugucgcuU...	16	1	Fm3
....................uccaggcacugucguucugucgcuA...	3	1	Fm3
....................uccaggcacugucguucugucgcuUu..	1	1	Fm3
....................uccaggcacugucguucugucgcuguaauc..	1	0	Fm3
.....................Ucaggcacugucguucugucg..	3	1	Fm3
.....................ccaggcacugucguucugucgc...	2	0	Fm3
.....................ccaggcacugucguucuCucgc...	1	1	Fm3
.....................Ucaggcacugucguucugucgc...	1	1	Fm3
.....................ccaggcacugucguucugucgcu..	6	0	Fm3
......................caggcacugucguucugu..	1	0	Fm3
......................caggcacugucguucuguc...	6	0	Fm3
......................caggcacugucguucugGcg..	1	1	Fm3
......................caggcacugucCuucugucg..	1	1	Fm3
......................caggcacugucguucugucU..	1	1	Fm3
......................caggcacugucguucuguAg..	1	1	Fm3
......................caggcacugucguuAugucg..	1	1	Fm3
......................caggcacugucguucugucg..	49	0	Fm3
......................caggcacugucguucugAcg..	1	1	Fm3
......................caggcacugucguucugucgU...	2	1	Fm3
......................caggcacugucguucugucgc...	4	0	Fm3
......................caggcacugucguucugucCc...	1	1	Fm3
......................caggcacugucguuUugucgc...	1	1	Fm3
......................caggcacugucguucugCcgcu..	1	1	Fm3
......................caggcacugucguucugucgcu..	6	0	Fm3
......................caggcacugucguucugucgUu..	3	1	Fm3
......................caggcacugucguucuguAgcu..	1	1	Fm3
.......................aggcacugucguucuguc...	5	0	Fm3
.......................Nggcacugucguucuguc...	1	1	Fm3
....cuguaaucguaaugugguagaaugaccuug..	1	0	Fm3
....uguaaucguaaugugguagaaugaccuug..	9	0	Fm3
....guagaaugaccuugccug....................................	9	0	Fm3
....guagaaugaccuugccuUa...................................	1	1	Fm3
....guagaaugacAuugccuga...................................	1	1	Fm3
....guagaaAgaccuugccuga...................................	1	1	Fm3
....guagaaugaccuugccGga...................................	2	1	Fm3
....guagaaugaccuugccuga...................................	63	0	Fm3
....guagUaugaccuugccuga...................................	1	1	Fm3
....guagaaugaccuugccugU...................................	4	1	Fm3
....guagaaugaccuugccugaG..................................	1	1	Fm3
....guagaaugaccuugccugau..................................	95	0	Fm3
....guaAaaugaccuugccugau..................................	1	1	Fm3
....guagaaugaccuugccGgau..................................	1	1	Fm3
....guagaaCgaccuugccugau..................................	1	1	Fm3
....guagaaugaccuugccuUau..................................	2	1	Fm3
....guagaaugGccuugccugau..................................	1	1	Fm3
....gGagaaugaccuugccugau..................................	1	1	Fm3
....guagaaugacGuugccugau..................................	1	1	Fm3
....guagaaugacUuugccugau..................................	1	1	Fm3
....guagaaugacAuugccugau..................................	2	1	Fm3
....guUgaaugaccuugccugau..................................	1	1	Fm3
....guagaaugaccuugccCgauc.................................	2	1	Fm3
....guagaaugacAuugccugauc.................................	1	1	Fm3
....guagaaugaccuugccugauU.................................	23	1	Fm3
....guagaaugaccuuUccugauc.................................	1	1	Fm3
....guagaaugaccuuAccugauc.................................	2	1	Fm3
....guagaaugaccCugccugauc.................................	1	1	Fm3
....guagaaugaccuugccGgauc.................................	3	1	Fm3
....guagaUugaccuugccugauc.................................	1	1	Fm3
....guagaaugaccuugccugauc.................................	567	0	Fm3
....guagaaugaccuugccAgauc.................................	2	1	Fm3
....guagaaugaccuugccugaCc.................................	1	1	Fm3
....guagaaugaccuugccuCauc.................................	2	1	Fm3

Mature Star

```
ucacugacggacucgugggguuccaggcacugucguucugucgcuguaaucguaauguguagaaugaccuugccugaucc...
```

```
ucacugacggacucgugggu uccaggcacugucguucugucg cuguaaucguaaugug guagaaugaccuugccugaucc ccaagggaacaucaacaucuggacacuugaac
..........................................................guagUaugaccuugccugauc.................................   1     1   Fm3
..........................................................guagaaugaccuugccuAauc.................................   4     1   Fm3
..........................................................guagaaugaccuugccugauA.................................   7     1   Fm3
..........................................................guagaaugaccuugccuUauc.................................   2     1   Fm3
..........................................................guagaaugaccuugccugauUc................................   7     1   Fm3
..........................................................guagaaugaUcuugccugaucc................................   2     1   Fm3
..........................................................Uuagaaugaccuugccugaucc................................   2     1   Fm3
..........................................................guagaaAgaccuugccugaucc................................   3     1   Fm3
..........................................................guagaaugacUuugccugaucc................................   11    1   Fm3
..........................................................guagaaugaccuugccugGucc................................   2     1   Fm3
..........................................................guagUaugaccuugccugaucc................................   4     1   Fm3
..........................................................guagaaugaccuugccAgaucc................................   12    1   Fm3
..........................................................guagaaugaccuGgccugaucc................................   1     1   Fm3
..........................................................guagaaugaccuugccuAaucc................................   22    1   Fm3
..........................................................guagaaugaccuugccGgaucc................................   7     1   Fm3
..........................................................guaAaaugaccuugccugaucc................................   2     1   Fm3
..........................................................guagaaugaccuuCccugaucc................................   2     1   Fm3
..........................................................guagaaugaccuugccuUaucc................................   20    1   Fm3
..........................................................Auagaaugaccuugccugaucc................................   1     1   Fm3
..........................................................guagaaugCccuugccugaucc................................   1     1   Fm3
..........................................................guagaaugaccuugccugaAcc................................   1     1   Fm3
..........................................................guagCaugaccuugccugaucc................................   4     1   Fm3
..........................................................guagaaugaccuugccugaucU................................   121   1   Fm3
..........................................................guagaaugaccuugccugauGc................................   1     1   Fm3
..........................................................guagaaugaccuugccugaucA................................   8     1   Fm3
..........................................................guagaaugaccCugccugaucc................................   2     1   Fm3
..........................................................guagaaugaccuugUcugaucc................................   4     1   Fm3
..........................................................guagaauUaccuugccugaucc................................   4     1   Fm3
..........................................................guagaaCgaccuugccugaucc................................   1     1   Fm3
..........................................................guagaaugaccuuAccugaucc................................   4     1   Fm3
..........................................................guagGaugaccuugccugaucc................................   1     1   Fm3
..........................................................guagaUugaccuugccugaucc................................   1     1   Fm3
..........................................................guagaaugaccuugccugauAc................................   1     1   Fm3
..........................................................guagaaugaccAugccugaucc................................   1     1   Fm3
..........................................................Nuagaaugaccuugccugaucc................................   2     1   Fm3
..........................................................guagaaugaccuugccugaCcc................................   1     1   Fm3
..........................................................guagaauAaccuugccugaucc................................   3     1   Fm3
..........................................................guagaaugacGuugccugaucc................................   11    1   Fm3
..........................................................guagaaugaccuugcUugaucc................................   4     1   Fm3
..........................................................guagaaugaccuugccugaucc................................   6160  0   Fm3
..........................................................gAagaaugaccuugccugaucc................................   3     1   Fm3
..........................................................guagaaugaccuugccuCaucc................................   26    1   Fm3
..........................................................guagaaugGccuugccugaucc................................   1     1   Fm3
..........................................................guagaaugaccuugccugCucc................................   3     1   Fm3
..........................................................guagaaugaccGugccugaucc................................   3     1   Fm3
..........................................................guagaaugaAcuugccugaucc................................   1     1   Fm3
..........................................................guaCaaugaccuugccugaucc................................   1     1   Fm3
..........................................................guagaaugaccuugcAugaucc................................   2     1   Fm3
..........................................................guagaaugacAuugccugaucc................................   14    1   Fm3
..........................................................guagaaugaccuugAcugaucc................................   3     1   Fm3
..........................................................guagaNugaccuugccugaucc................................   2     1   Fm3
..........................................................guagaaugaGcuugccugaucc................................   1     1   Fm3
..........................................................guagaaugaccuugccCgaucc................................   18    1   Fm3
..........................................................gCagaaugaccuugccugaucc................................   2     1   Fm3
..........................................................guUgaaugaccuugccugaucc................................   1     1   Fm3
..........................................................guGgaaugaccuugccugaucc................................   1     1   Fm3
..........................................................guagaaugaccuCgccugaucc................................   3     1   Fm3
..........................................................guagaaugUccuugccugaucc................................   3     1   Fm3
..........................................................guagaaugaccuuUccugaucc................................   3     1   Fm3
..........................................................guagaaugaccuugccugauccU...............................   1295  1   Fm3
..........................................................guagaaugaccuugccugauccc...............................   2     0   Fm3
..........................................................guagaaugaccuugccugauccA...............................   337   1   Fm3
..........................................................guagaaugaccuugccugauccG...............................   2     1   Fm3
..........................................................guagaaugaccuugccugaucccU..............................   1     1   Fm3
..........................................................guagaaugaccuugccugauccUc..............................   1     1   Fm3
...........................................................uagaaugaccuugccugau...................................   6     0   Fm3
...........................................................uagaaugaccuugccugauc..................................   8     0   Fm3
...........................................................uagaaugaccuugccugaucc.................................   8     0   Fm3
...........................................................uagaaugaccuugccugGucc.................................   1     1   Fm3
...........................................................uagaaugaccuugccugauAc.................................   1     1   Fm3
```

```
                    Mature                                Star
ucacugacggacucgugggu uccaggcacugucguucugucg cuguaaucguaaugug guagaaugaccuugccugaucc ccaagggaacaucaacaucuggacacuugaac
...........................................................uagaaugaccuugccugauccU...............................  17    1  Fm3

...................Cuccaggcacugucguucuguc.......................................................................  1     1  Mm3
...................Cuccaggcacugucguucugucg......................................................................  3     1  Mm3
....................uccaggcacugucguucu..........................................................................  27    0  Mm3
....................uccaggcacugucguucuU.........................................................................  9     1  Mm3
....................uccaggcacugucguucug.........................................................................  19    0  Mm3
....................uccaggcacuguUguucug.........................................................................  1     1  Mm3
....................uccaggcacuAucguucugu........................................................................  1     1  Mm3
....................uccaggcacugucguucugA........................................................................  8     1  Mm3
....................uccaggcacugucguucugu........................................................................  185   0  Mm3
....................Cccaggcacugucguucugu........................................................................  1     1  Mm3
....................uccaggcacugucguucCgu........................................................................  1     1  Mm3
....................uccaggcacuUucguucugu........................................................................  1     1  Mm3
....................uccaggcacugucguucAguc.......................................................................  3     1  Mm3
....................uccaggcacugucguucCguc.......................................................................  4     1  Mm3
....................uccaggcacugucguuGuguc.......................................................................  2     1  Mm3
....................ucUaggcacugucguucuguc.......................................................................  1     1  Mm3
....................ucGaggcacugucguucuguc.......................................................................  1     1  Mm3
....................uccaggcaUugucguucuguc.......................................................................  2     1  Mm3
....................uccaggcacuAucguucuguc.......................................................................  1     1  Mm3
....................uccaAgcacugucguucuguc.......................................................................  1     1  Mm3
....................uccaggcacugucguuAuguc.......................................................................  4     1  Mm3
....................uAcaggcacugucguucuguc.......................................................................  1     1  Mm3
....................uccaggcacugucguucuguc.......................................................................  632   0  Mm3
....................uccaggcacugucguucuguA.......................................................................  13    1  Mm3
....................uccaggcacuCucguucuguc.......................................................................  1     1  Mm3
....................uUcaggcacugucguucuguc.......................................................................  1     1  Mm3
....................uccaggcacugucguucuguU.......................................................................  11    1  Mm3
....................uccaggcacugucguucugAcg......................................................................  1     1  Mm3
....................uccagNcacugucguucugucg......................................................................  1     1  Mm3
....................uccaggcacugucgAucugucg......................................................................  1     1  Mm3
....................ucAaggcacugucguucugucg......................................................................  1     1  Mm3
....................Nccaggcacugucguucugucg......................................................................  2     1  Mm3
....................uccaggcacuAucguucugucg......................................................................  15    1  Mm3
....................ucUaggcacugucguucugucg......................................................................  2     1  Mm3
....................uccaggcacugucguuUugucg......................................................................  14    1  Mm3
....................uccaggcUcugucguucugucg......................................................................  1     1  Mm3
....................uccaggcacugucguucugGcg......................................................................  1     1  Mm3
....................uccaggcacCgucguucugucg......................................................................  1     1  Mm3
....................uccaggcacugucguucugucA.....................................................................  42    1  Mm3
....................uccaggcacugucguuAugucg......................................................................  37    1  Mm3
....................uccaggcacugucguucuAucg......................................................................  1     1  Mm3
....................uccaggcacugucguuGugucg......................................................................  7     1  Mm3
....................uccaggcacugucgCucugucg......................................................................  1     1  Mm3
....................uccaggcacugucguucAgucg......................................................................  40    1  Mm3
....................uccaggcacugucguCcugucg......................................................................  1     1  Mm3
....................uccaggcacuUucguucugucg......................................................................  6     1  Mm3
....................uccaggcacugucguucugucg......................................................................  7405  0  Mm3
....................uccaggcacGgucguucugucg......................................................................  1     1  Mm3
....................uccaggcacugucguucugucU.....................................................................  112   1  Mm3
....................uAcaggcacugucguucugucg......................................................................  6     1  Mm3
....................Cccaggcacugucguucugucg......................................................................  3     1  Mm3
....................uGcaggcacugucguucugucg......................................................................  2     1  Mm3
....................uUcaggcacugucguucugucg......................................................................  3     1  Mm3
....................uccGggcacugucguucugucg......................................................................  3     1  Mm3
....................uccaggcacugucCuucugucg......................................................................  1     1  Mm3
....................uccaggcacuguUguucugucg......................................................................  5     1  Mm3
....................uccaggcacugucguucGgucg......................................................................  37    1  Mm3
....................uccagUcacugucguucugucg......................................................................  2     1  Mm3
....................uccaggcacugucguucugCcg......................................................................  3     1  Mm3
....................uccaggcacuCucguucugucg......................................................................  8     1  Mm3
....................uccagAcacugucguucugucg......................................................................  1     1  Mm3
....................uccaUgcacugucguucugucg......................................................................  2     1  Mm3
....................uccaggUacugucguucugucg......................................................................  3     1  Mm3
....................uccaggcacugucguucugucC.....................................................................  2     1  Mm3
....................uccaggcacugucguucuguUg......................................................................  1     1  Mm3
....................uccaggcacugucgGucugucg......................................................................  2     1  Mm3
....................uccaggcacugucguucCgucg......................................................................  45    1  Mm3
....................uccaggcacugucAuucugucg......................................................................  2     1  Mm3
```

Mature Star

```
ucacugacggacucgugggguuccaggcacugucguucugucgcuguaaucguaaugugguagaaugaccuugccugaucccaagggaacaucaacaucuggacacuugaac
....................uccaggcacugAcguucugucg......................................................................  2     1  Mm3
....................uccaCgcacugucguucugucgc.....................................................................  1     1  Mm3
....................uccaggcacugucguucCgucgc.....................................................................  2     1  Mm3
....................uccaggcacugucguucugucgG.....................................................................  8     1  Mm3
....................uccaggcacugucguucugucgU.....................................................................  250   1  Mm3
....................uccaggcacugucguuAugucgc.....................................................................  1     1  Mm3
....................uccaggcacugucguucugucgA.....................................................................  274   1  Mm3
....................uccaggNacugucguucugucgc.....................................................................  1     1  Mm3
....................uccaggcacugucguucugucgc.....................................................................  561   0  Mm3
....................uccaggcacugucguucGgucgc.....................................................................  6     1  Mm3
....................uccaggcacugucguucAgucgc.....................................................................  7     1  Mm3
....................Nccaggcacugucguucugucgc.....................................................................  1     1  Mm3
....................uccaggcUcugucguucugucgc.....................................................................  1     1  Mm3
....................uccaggcacuCucguucugucgc.....................................................................  2     1  Mm3
....................uccaggcacuUucguucugucgc.....................................................................  1     1  Mm3
....................uAcaggcacugucguucugucgc.....................................................................  1     1  Mm3
....................uccaggcacuAucguucugucgc.....................................................................  1     1  Mm3
....................uccCggcacugucguucugucgc.....................................................................  1     1  Mm3
....................uccaggcacugucgCucugucgc.....................................................................  1     1  Mm3
....................uccaggcacugucguucAgucgcu....................................................................  1     1  Mm3
....................uccaggcacugucguucugucgAu....................................................................  4     1  Mm3
....................uccaggcacugucguucugucgcu....................................................................  129   0  Mm3
....................uccCggcacugucguucugucgcu....................................................................  1     1  Mm3
....................uccaggcacugucguucGgucgcu....................................................................  1     1  Mm3
....................uccaggcacugucguuGugucgcu....................................................................  2     1  Mm3
....................uccaggcacugucguucugucgUu....................................................................  89    1  Mm3
....................uccaggcacugucguucugucgcC....................................................................  2     1  Mm3
....................uccaggcacugucguucugucgcG....................................................................  5     1  Mm3
....................uccaggcacugucguucugucgcA....................................................................  41    1  Mm3
....................uccaggcacugucguucCgucgcu....................................................................  1     1  Mm3
....................uccaggcacugucguucugucgcuU...................................................................  12    1  Mm3
....................uccaggcacugucguucugucgcuUu..................................................................  1     1  Mm3
....................uccaggcacugucguucugucgcuAu..................................................................  1     1  Mm3
....................uccaggcacugucguucGgucgcuguaauc..............................................................  1     1  Mm3
....................uccaggcacugucguucugucgcuguaauc..............................................................  7     0  Mm3
.....................ccaggcacugucguucugucg......................................................................  2     0  Mm3
.....................ccaggcacugucguucugucgc.....................................................................  2     0  Mm3
......................caggcacugucguucuguU.......................................................................  1     1  Mm3
......................caggcacugucguucugucg......................................................................  22    0  Mm3
......................cagCcacugucguucugucg......................................................................  1     1  Mm3
......................caggcacugucguucugCcg......................................................................  1     1  Mm3
......................caggcacugucguucugucgc.....................................................................  5     0  Mm3
......................caggcacugucguucugucgU.....................................................................  1     1  Mm3
......................caggcacugucguucugucgA.....................................................................  1     1  Mm3
......................caggcacugucguucugucgUu....................................................................  1     1  Mm3
.......................aggcacugucguucuguc.......................................................................  1     0  Mm3
.......................aggcacugucguucugucg......................................................................  1     0  Mm3
.......................aggcacugucguucugucgcu....................................................................  2     0  Mm3
..........................................cuguaaucguaaugugguagaaugaccu..........................................  1     0  Mm3
...........................................uguaaucguaaugugguagaaugaccuugcc......................................  1     0  Mm3
...........................................uguaaucguaaugugguagaaugaccuugccugaucc................................  2     0  Mm3
..........................................................guagaaugaccuugccug....................................  5     0  Mm3
..........................................................guagaaugaccuugccugU...................................  1     1  Mm3
..........................................................guagaaugaccuugccuga...................................  5     0  Mm3
..........................................................guagaaugaccuugccugau..................................  18    0  Mm3
..........................................................guagaaugaccuugccuAau..................................  1     1  Mm3
..........................................................guagaaugaccuugccugauA.................................  1     1  Mm3
..........................................................guagaaugaccuugccugauU.................................  2     1  Mm3
..........................................................guagaaugacAuugccugauc.................................  1     1  Mm3
..........................................................guagaaugaccuugccugauc.................................  110   0  Mm3
..........................................................guagaaCgaccuugccugauc.................................  1     1  Mm3
..........................................................guagaaugaccuugUcugaucc................................  1     1  Mm3
..........................................................guaAaaugaccuugccugaucc................................  1     1  Mm3
..........................................................Nuagaaugaccuugccugaucc................................  1     1  Mm3
..........................................................guagaaugaccuugccCgaucc................................  5     1  Mm3
..........................................................guagaaNgaccuugccugaucc................................  1     1  Mm3
..........................................................guagaaugaccuugccugaucc................................  1709  0  Mm3
..........................................................guagaaugacGuugccugaucc................................  2     1  Mm3
..........................................................guGgaaugaccuugccugaucc................................  1     1  Mm3
..........................................................gCagaaugaccuugccugaucc................................  1     1  Mm3
```

Mature Star

ucacugacggacucgugggu**uccaggcacugucguucugucg**cuguaaucguaaugug**guagaaugaccuugccugaucc**ccaagggaacaucaacaucuggacacuugaac			
....guagaaugacAuugccugaucc................................	2	1	Mm3
....guagaaugaccuugccAgaucc................................	2	1	Mm3
....guagCaugaccuugccugaucc................................	3	1	Mm3
....guagaaugaccuugccuCaucc................................	11	1	Mm3
....guagaaugaccCugccugaucc................................	1	1	Mm3
....guagaaugaccuugccuAaucc................................	5	1	Mm3
....guagaauUaccuugccugaucc................................	1	1	Mm3
....Uuagaaugaccuugccugaucc................................	2	1	Mm3
....guagaaugUccuugccugaucc................................	1	1	Mm3
....guagGaugaccuugccugaucc................................	1	1	Mm3
....guagaaugaccuugccugGucc................................	1	1	Mm3
....guagaaugacUuugccugaucc................................	1	1	Mm3
....guagaaugaccuugccuUaucc................................	6	1	Mm3
....guagaaugaccuugccugaucU................................	22	1	Mm3
....guagUaugaccuugccugaucc................................	2	1	Mm3
....guagaaugaccuugccugauAc................................	2	1	Mm3
....guagaaAgaccuugccugaucc................................	1	1	Mm3
....guagaaugaccuugccugaucA................................	1	1	Mm3
....guagaaugaccuugccugauccG...............................	8	1	Mm3
....guagaaugaccuugccugauccA...............................	92	1	Mm3
....guagaaugaccuugccugauccU...............................	387	1	Mm3
....guagaaugaccuugccugauccc...............................	2	0	Mm3
....uagaaugaccuugccugaucc................................	3	0	Mm3
....uagaaugaccuugccugauccU...............................	3	1	Mm3
